# Dual inhibition of glycolysis and glutaminolysis for synergistic therapy of rheumatoid arthritis

**DOI:** 10.1186/s13075-023-03161-0

**Published:** 2023-09-20

**Authors:** Shanzay Ahmed, Christopher B. Mahony, Alyssa Torres, Jessica Murillo-Saich, Samuel Kemble, Martha Cedeno, Peter John, Attya Bhatti, Adam P. Croft, Monica Guma

**Affiliations:** 1 Department of Healthcare Biotechnology, Atta-Ur-Rahman School of Applied Biosciences (ASAB), National University of Sciences and Technology (NUST), Islamabad, 44000 Pakistan; 2 Department of Medicine, Division of Rheumatology, Allergy and Immunology, University of California, San Diego, 9500 Gilman Drive, La Jolla, CA USA; 3 Rheumatology Research Group, Institute of Inflammation and Ageing, Queen Elizabeth Hospital, University of Birmingham, Birmingham, UK

**Keywords:** Rheumatoid arthritis, Fibroblast-like synoviocytes, Glucose metabolism, Glutamine metabolism hexokinase, Glutaminase

## Abstract

**Background:**

Synovial fibroblasts in rheumatoid arthritis (RAFLS) exhibit a pathological aberration of glycolysis and glutaminolysis. Henceforth, we aimed to investigate if dual inhibition of these pathways by phytobiological compound c28MS has the potential of synergistic therapy for arthritis by targeting both glucose and glutamine metabolism.

**Methods:**

The presence of HK2 and GLS across various cell types and associated gene expression in human synovial cells and a murine model of arthritis was evaluated by scRNA-seq. The metabolic profiling of RAFLS cells was done using H^1^-nuclear magnetic resonance spectroscopy under glycolytic and glutaminolytic inhibitory conditions by incubating with 3-bromopyruvate, CB839, or dual inhibitor c28MS. FLS functional analysis was conducted under similar conditions. ELISA was employed for the quantification of IL-6, CCL2, and MMP3. K/BxN sera was administered to mice to induce arthritis for in vivo arthritis experiments.

**Results:**

scRNA-seq analysis revealed that many fibroblasts expressed *Hk2* along with *Gls* with several genes including *Ptgs2*, *Hif1a*, *Timp1*, *Cxcl5*, and *Plod2* only associated with double-positive fibroblasts, suggesting that dual inhibition can be an attractive target for fibroblasts. Metabolomic and functional analysis revealed that c28MS decreased the aggressive behavior of RAFLS by targeting both upregulated glycolysis and glutaminolysis. c28MS administered in vivo significantly decreased the severity of arthritis in the K/BxN model.

**Conclusion:**

Our findings imply that dual inhibition of glycolysis and glutaminolysis could be an effective approach for the treatment of RA. It also suggests that targeting more than one metabolic pathway can be a novel treatment approach in non-cancer diseases.

**Supplementary Information:**

The online version contains supplementary material available at 10.1186/s13075-023-03161-0.

## Introduction

Rheumatoid arthritis (RA) is a systemic autoimmune disease of the synovial joints. The disorder is best described by swelling, pain, and stiffness of the affected joints thus negatively impacting the life of the affected individuals [1]. The primary location of inflammation is the lining of the membrane of the synovium. Normal fibroblast-like synoviocytes (FLS) adopt a more aggressive phenotype during the pathogenesis of RA. The altered phenotype is more aggressive due to an increase in the production of chemokines, cytokines, and bone and cartilage degrading enzymes [2–4]. Increased invasive and migrative capability is driven by metabolic reprogramming of the RAFLS. Prior metabolomics studies elucidated that a disrupted metabolism is closely associated to the pathology of RA [5, 6]. This is a probable consequence of the augmented biosynthetic and bioenergetic requisites of chronic inflammation coupled with shifts in the availability of nutrition and oxygen for tissues during inflammation [7, 8]. Previous studies have also indicated that metabolic pathways can be novel therapeutic targets for RA [9].

Glucose and glutamine are two predominant nutrients in the cell that provide energy and biomass for cellular growth and proliferation. The breakdown of glucose and glutamine serves as an essential source of carbon intermediates required for the assembly of various macromolecules. In case of the suppression of glucose metabolism, glutamine may serve as a substitute source for energy and biomass [10]. Thus, the inhibition of a single enzyme from the glycolysis or glutaminolysis pathways might not be as efficient therapeutically as dual inhibition of target enzymes. Of note, several studies showed that glycolysis and glutaminolysis are upregulated in RA [8, 9, 11].

At present, the current regimen of medicines for RA includes NSAIDs (nonsteroidal anti-inflammatory drugs), DMARDS (disease-modifying anti-rheumatic drugs), and glucocorticoids. Despite recent improvements in treatment and disease management for RA, existing medications do not fully target the dysregulated functioning of FLS [12]. Among the most common compounds being employed for the inhibition of glycolysis and glutaminolysis are CB839 and 3-bromopyruvate (3BrPy), respectively [9, 13]. While CB839 is a selective inhibitor and is being rigorously explored for its therapeutic potential in clinical trials [9, 14, 15], 3BrPy had limited success in this context [16]. Furthermore, despite HK2 being one of the 3BrPy targets, 3BrPy is known to affect several other enzymes of the glycolysis and TCA cycle [16–19]. This stems an opportunity for the exploration of new and improved therapeutics to either replace or complement existing therapies.

In our previous study [20], we discovered novel compounds with the potential of dual inhibition of key enzymes of glycolysis and glutaminolysis, i.e., HK2 and GLS. Computational assessment revealed that 28MS inhibits both target enzymes similarly. Hence, the purpose of the undertaken study was in vitro and in vivo evaluation of the therapeutic efficiency of this compound to determine whether dual inhibition is a better approach to treat RA.

## Methods

### Reagents

Tumor necrosis factor (TNF, 10 ng/ml) was acquired from R&D Systems Inc. CB-839 (300 nM) was purchased from Selleck, 3Bromopyruvate (3BrPy) from Sigma Aldrich, and compound 28MS (c28MS) from Chembridge Corporation.

### Single-cell RNA sequencing from serum transfer-induced arthritis (STIA) model

All animal protocols were authorized by the UK Home Office and implemented in accordance with the UK’s Animals (Scientific Procedures) Act of 1986 and the UK Home Office Code of Practice. The research project and all the protocols were permitted by the University of Birmingham Animal Ethics Review Committee who provisioned ethical surveillance of the study. The C57BL/6 mice were obtained from Charles River. All mice utilized for experimental procedures were 8–10-week-old male mice. Single animals were regarded as experimental entities. Induction of STIA was done by intravenous injection consisting of 100 μl of serum derived from KRN mice (K/BxN) [21]. Joint thickness of the wrist or ankle was examined using calipers and described as a variation from the baseline. The acuteness of swelling of the joint was calculated employing the AUC analysis of sequential measurements. Complete joints were dissected and transferred into RPMI-1640 (+ 2% FCS) having collagenase D (Roche) 0.1 g/ml and 0.01 g/ml of DNAse I (Sigma-Aldrich). Obtained samples were then incubated for 40 min at 37 °C. This was followed by incubation for 20 min with a medium incorporated with 0.1 g/ml collagenase dispase (Roche) and 0.01 g/ml DNase I. Synovial CD45 negative cells obtained from hind limbs at day 0, day 7 to 9 peak, day 15 resolving, or day 22 resolved, of STIA murine joints (biological replicates *n* = 3, each was made from of cells extracted from the joints of three different animals) were sorted and subsequently purified using the BD ARIA and finally acquired with the 10 × Genomics Chromium system. Murine data is available at GSE230145.

Analyses of 3’-scRNAseq were completed as previously described [22], using the same software, functions, and QC thresholds. Further subcluster analysis of the STIA fibroblast subset was completed using FindNeighgbours(), FindClusters() (From Seurat (v4.0.3)). Subcluster identity was assigned based on the top expressing genes calculated using FindAllMarkers(). Human data was obtained from ImmPort (https://www.immport.org/shared/study/SDY998). QC metrics used were genes_detected > 200 and genes_detected < 5000 percent_mt_molecules < 0.25. Full analysis code is available at chrismahony/Ahmed-et-al.-2023 (github.com).

### Human FLS

FLS were obtained from the affected joints of patients diagnosed with RA and undergoing entire joint replacement as described by [23]. RAFLS were cultured in DMEM with 25 mM of glucose (Dulbecco’s modified Eagle’s medium), not including sodium pyruvate, and supplemented with 10% of the fetal bovine serum (FBS), 2 mM L-glutamine, 100 units/ml penicillin, and 100 μg/ml streptomycin (glucose medium). Experiments involving the glutamine dictating conditions (free/low-glucose) were performed with dialyzed FBS (no. A3382001; Gibco) in DMEM without glucose, glutamine, phenol red, or sodium pyruvate (no. A1443001; Gibco). DMEM without glucose and glutamine was supplemented with 6 mM of glutamine for all the experiments involving glucose-free media and 2 mM glucose and 6 mM of glutamine for all experiments using low-glucose media.

### Western blot

RAFLS were treated with compound 28MS and vehicle for 24 or 48 h in glucose or glucose-free media and then pelleted. Total cellular lysates were made by employing ice-cold lysis buffer (0.5 ml of Tris1M (pH7.6), 1.4 ml of 5 M NaCl, 5 ml of NP40 1%, 0.5 ml of 0.5 M EDTA, 0.5 ml of 0.5 mM NaF, 0.2 ml of 0.1 mM Na Orthovanadate, up to 50 ml distilled water), supplemented with protease inhibitors. The concentration of the protein was determined by conducting a Bradford Assay. Fifteen micrograms of protein was loaded and separated on a TGX-gel (BIO-RAD, Cat#4,569,036) and consequently transferred to a suitable PVDF membrane. Membrane blocking was done with 5% skim milk and thereafter incubated with primary antibodies in 5% milk at 4 °C with primary antibodies recognizing HK2 (1:500, Santa Cruz Biotechnology, USA) GLS1 (1:1000, Abcam, USA) and housekeeping alpha Tubulin (1:1000, Sigma, USA). The following day the membrane was then incubated with the appropriate secondary antibody (1:2000) for a period of 2 h at r.t.p (room temperature). Western ECL substrate, a chemiluminescence detection reagent, was used to visualize and detect proteins of interest.

### H^1^-Nuclear Magnetic Resonance (NMR) spectroscopy, acquisition, and processing of spectra

Approximately 600,000 to 800,000 RAFLS were plated and placed in an incubator at 37 °C for 24 h. 1X PBS was used to wash the cells the following day. Appropriate treatment was added to the cells in 10% FBS medium under high (25 mM glucose and 6 mM glutamine) or low-glucose (2 mM glucose and 6 mM glutamine) conditions and incubated again at 37 °C for 24 h. Ensuing this, cells and supernatant were collected for metabolite extraction as described by [8]. The NMR spectra were recorded employing a 600-MHz Bruker Avance III NMR spectrometer, attached to a 1.7-mm triple resonance cryoprobe. The data were collected at the NMR facility at UC San Diego, Skaggs School of Pharmacy and Pharmaceutical Sciences. The obtained metabolites were identified and quantified using Chenomx-NMR Suite 9. The software consists of a built-in reference library to identify the peak of the metabolites as per their chemical shifts. The concentration of the metabolites was obtained in accordance with TSP-d4 (# 269,913; Millipore Sigma), used as an internal standard in this experiment. Metabolite concentrations obtained from the pellets were normalized corresponding to their cell count. On the other hand, metabolite concentrations from the supernatant were calculated after deduction from the values of the control in complete DMEM. Negative values would imply that the particular metabolite had been utilized by the respective cells in the culture medium (concentrations of the metabolites are presented in μM).

### Cell viability

Three thousand RAFLS were seeded in triplicates in a 96-well plate. Appropriate conditions were added in glucose (25 mM glucose and 6 mM glutamine) and low-glucose (2 mM glucose and 6 mM glutamine) mediums and incubated for 24 h at 37 °C. The following day 10% MTT dye was added to the wells and dissolved in 200 μl of DMSO after 4 h of incubation. Cell density was recorded at 550 and 690 nm.

### Matrigel invasion

RAFLS were disassociated using 0.05% trypsin and consequently centrifuged at 1200 rpm for 5 min to pellet the cells. ~ 1.1 × 10^6^ to 1.2 × 10^6^ cells were washed with media and repelleted. The repelleted cells were then resuspended in 45 μL of matrigel (BD Biosciences, #356,231) and 45 μL of media with no serum. Five microliters of this cell mixture was used to make spheroids. The spheroids were then placed in an incubator at 37 °C for 9 min to settle and solidify. Appropriate treatments were added to the cells under high glucose (25 mM) or glucose-free conditions in 1% or 10% FBS and incubated for 24 h at 37 °C. Following incubation, the spheroids were fixed by the addition of 4% paraformaldehyde (PFA). The ensuing staining of the spheroids was done using 0.05% crystal violet and imaged at the microscope facility (UCSD School of Medicine Microscopy Core) with a Keyence Fluorescence Microscope. Image J (https://imagej.nih.gov/ij/) was used for the quantification of the invaded area.

### Migration scratch assay

One hundred fifty thousand RAFLS were seeded in a 6-well plate and starved with 0.1% FBS overnight to circumvent proliferation and subsequently scratched using a sterile tip of the pipette to create two cross-sections. The wells were then washed with 1 × PBS. Appropriate treatments were added to cells under high glucose (25 mM) or glucose-free conditions in 1% or 10% FBS and incubated for 24 h at 37 °C to allow them to migrate. A decrease in length indicates an increased migration rate into the wound. Cells were then fixed using the 4% paraformaldehyde (PFA) solution, stained afterwards with 0.05% crystal violet, and imaged at the microscope facility (UCSD School of Medicine Microscopy Core) with a Keyence Fluorescence Microscope. Image J (https://imagej.nih.gov/ij/) was used for the quantification of the wound margins at both cross-sections by measuring the distance between the cells on either side of the made scratches.

### MTT assay

Three thousand RAFLS cells were used as seed per well in a 96-multi-well plate. Three replicate wells were established for each condition. The plated cells were starved overnight with 0.1% FBS to prevent proliferation. Cells were washed with 1X PBS and appropriate conditions were added under high (25 mM glucose and 6 mM glutamine) or low-glucose (2 mM glucose and 6 mM glutamine) conditions in 1% or 10% FBS. RA FLS were then allowed to proliferate at 37 °C. On day 5 after the addition of conditions, 10% MTT dye was added to the wells with appropriate conditions. After an incubation period of 4 h crystals were subsequently dissolved in 200 μL DMSO. Ensuing this the optical density was determined at 550 nm and 690 nm with replicates being averaged for each condition.

### EDU proliferation assay

The Click-iT** ®** Plus Edu Imaging Test (Life technologies) kit was used to perform the EdU proliferation assay as per the manufacturer’s instructions. Seven thousand RAFLS cells were plated on glass coverslips placed at the bottom of every well of a 24 multi-well plate. The cells were starved overnight with 0.1% FBS, and the following day, cells were cultured under high (25 mM glucose and 6 mM glutamine) or low-glucose (2 mM glucose and 6 mM glutamine) conditions in 1% or 10% FBS. EdU was added to the cells 24 h after the addition of conditions at a concentration of 10 uM. On the fifth day after the addition of conditions, cells were fixed with 4% PFA, counterstained with Hoechst, and consequently mounted on slides. EdU-positive cells were counted in Hoechst positive for each condition. The pictures of three random areas on each slide were obtained at a microscope facility (UC San Diego School of Medicine Microscopy Core) with a Keyence Fluorescence Microscope.

#### ELISA

Ninety-six multi-well plate was used for seeding 10,000 RAFLS for performing ELISA. For each condition, 3 replicate wells were established. The inhibitors were added under high glucose (25 mM) or glucose-free conditions 1 h prior to TNF alpha stimulation (5 ng/ml) and incubated for 24 h at 37 °C. The next day, the supernatant was aspired and IL-6, MMP3, and CCL2 (R&D systems) were measured as per the manufacturer’s instructions.

### Serum transfer-induced arthritis (STIA) inhibition with c28MS compound

KRN-T cell receptor–transgenic mice were obtained as a gift from Dr. D. Mathis (Harvard Medical School, Boston, MA) and Dr. C. Benoist (Institut de Génétique et de Biologie Moléculaire et Cellulaire, Strasbourg, France). For serum transfer, the sera from grownup arthritic K/BxN mice were combined. 150 μl of K/BxN arthritic serum was injected intraperitoneally in 3 to 4 months old, female C57BL/6 mice. Mice were treated with vehicle and compound 28MS(2.5 mg/kg) from day 0 to 10. Clinical scores were determined in the receiver mice post-serum transfer, as described previously [24]. On day 10 after the injection of serum, the mice were euthanized, and their paws were collected for histopathology. Furthermore, the effect of c28MS was also compared to the standard drug methotrexate (10 mg/kg daily) in male C57BL/6 mice to assess the efficacy of the compound against a standard drug. Clinical score and joint swelling were recorded and taken as a measure to review the efficacy of standard treatment as opposed to alternative treatment.

### Histopathology

Collected joints from female C57BL/6 were then fixed by employing a formalin fixative, decalcified in CalEx for 24 h, and consequently, paraffin-embedded. The embedded sections of the paw were then stained with dyes hematoxylin and eosin (H&E) and Safranin O [24].

### Statistical analysis

GraphPad Prism software version 9 was used for statistical analysis. The normality of the data was determined using a D’Agostino-Pearson or Shapiro–Wilk test for normality. Depending upon the normality of the obtained data, Student’s *T*-test or Mann–Whitney were employed to compare between 2 groups. For the analysis of more than two variables, ANOVA (ordinary one-way analysis of variance) or Kruskal–Wallis test was utilized as per the normality of the distribution ensued by Tukey’s or Dunn’s multiple comparisons, as respective post hoc analysis tests. Results were considered significant if the 2-sided *p*-value was less than 0.05. Heatmaps of metabolites were created by using “gplots” package (https://cran.r-project.org/web/packages/gplots/gplots.pdf) in R.

## Results

### Hexokinase- and glutaminase-driven differential gene expression

**Fig. 1:**
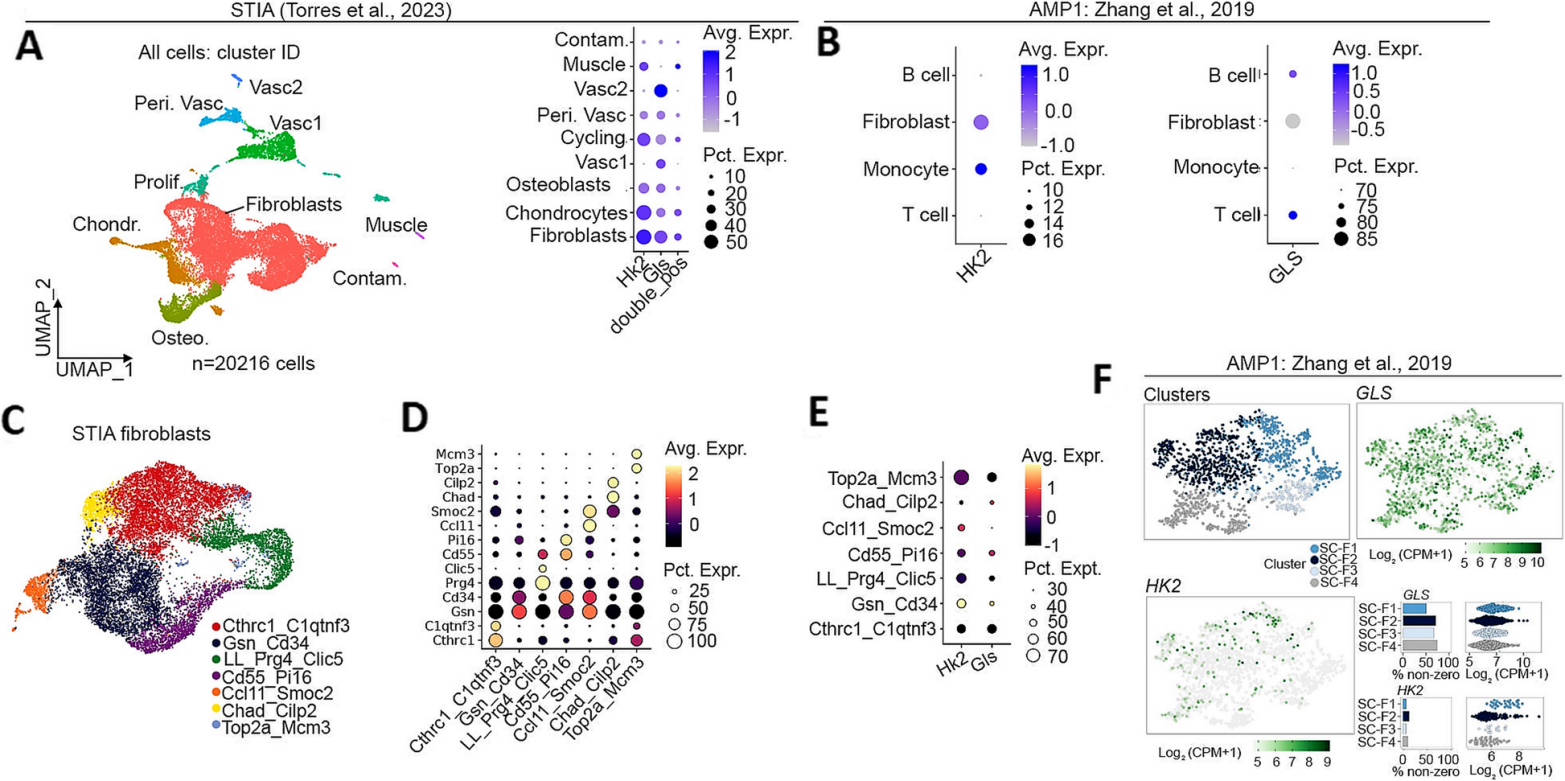
HK2 and GLS are associated with specific synovial fibroblast subsets. **A** scRNA-seq UMAP of all cells sequenced from Torres et al. (2023) [22] along with expression of *Hk2*, *Gls*, and the signature of both genes. **B** Expression of HK2 and GLS in publicly available data from Zhang et al. [25]. **C**, **D** Establishment of STIA fibroblast subclusters. **E** Expression of *Gls* and *Hk2* in STIA fibroblast subclusters. **F** Expression of GLS and HK2 in previously defined fibroblast sub-cluster in human data from [25]

We first wanted to establish which cells across the murine synovial stroma express both *Hk2* and *Gls*. To achieve this, we studied scRNA-seq data from digested synovial tissue from the hind limbs of mice at different timepoints of the STIA disease stage (rest: day 0, peak: day 7–9, resolving: day 15, resolved: day 22) and then completed 3’ mRNA sequencing which produced a total of 20,216 cells that passed QC (Fig. 1A) [22]. The main cell types were then annotated (Fig. 1A) based on marker gene expression as in Torres et al. (2023) [22] and identified that many fibroblasts expressed *Hk2* along with *Gls* (although expression of *Gls* was also noted on vascular cells), which was confirmed by also plotting the gene signature from both genes (Fig. 1A). We then verified these findings in human synovial cells from publicly available data [25], which showed HK2 to be mainly expressed in fibroblasts and monocytes and GLS to be mainly expressed in Fibroblasts and T cells (Fig. 1B). As different subtypes of fibroblasts have been identified to be functionally different in human and murine synovial tissues, we established murine fibroblast subclusters based on marker gene expression (Fig. 1C, D). We then plotted HK2 and GLS expression in the different subtypes of fibroblasts in murine (Fig. 1E) and established human (Fig. 1F) fibroblast subsets. *Hk2* and *Gls* showed co-expression in Gsn_Cd34 murine fibroblasts (Fig. 1E). This was corroborated by human data from Zhang et al. (2019) where HK2 and GLS are both expressed more highly in their SC-F1 cluster (a CD34 positive cluster) (Fig. 1F), despite the overall weaker expression of HK2.

**Fig. 2:**
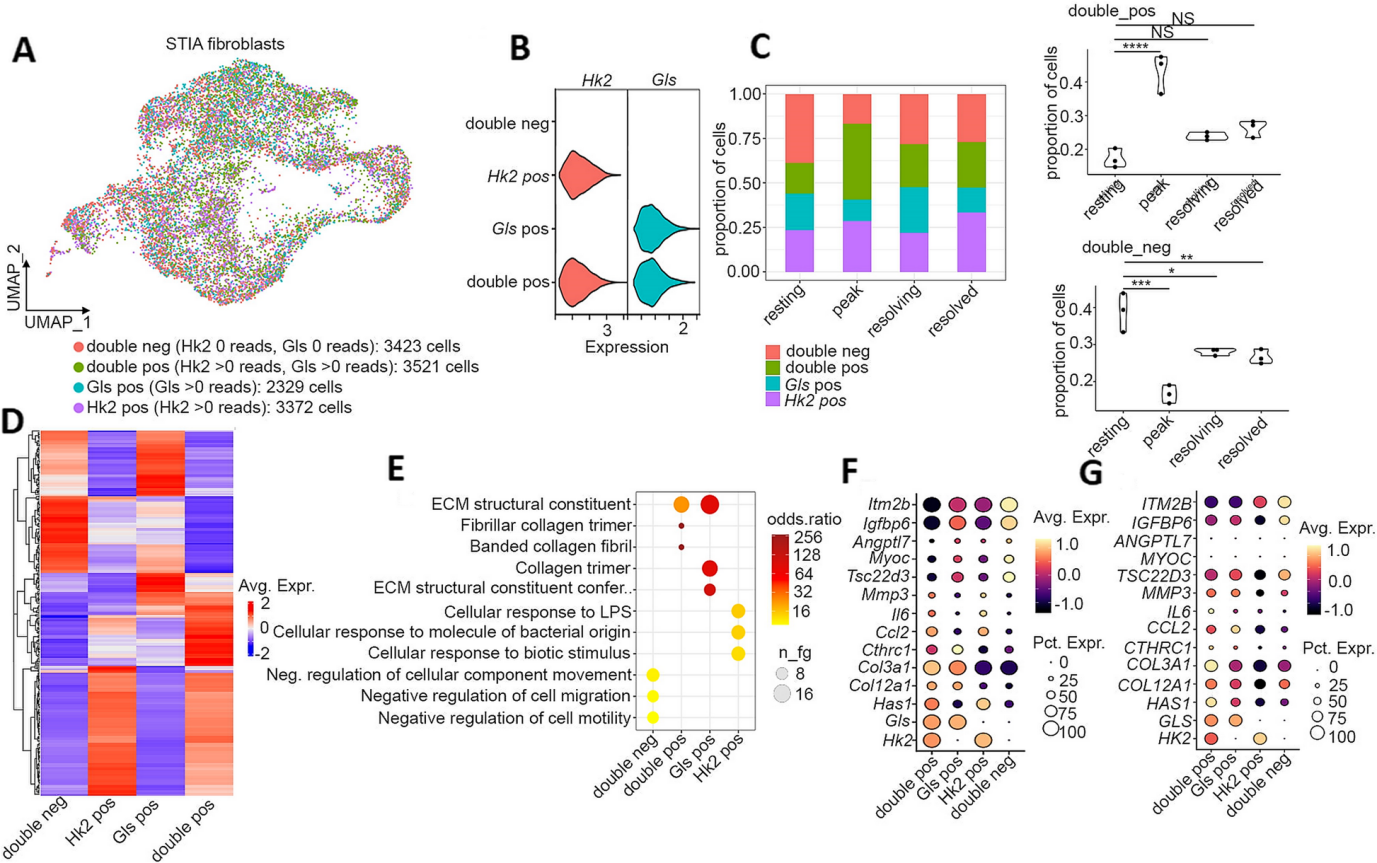
Expression of HK2 and GLS is associated with differential gene expression in fibroblasts. **A**, **B** Segregation of synovial STIA fibroblasts into *Gls*-positive, *Hk2*-positive, double-negative, and double-positive. **C** Proportion of *Gls*-positive, *Hk2*-positive, double-negative, and double-positive cells in STIA fibroblasts. Each dot represents a sample. Statistical significance was completed using one-way ANOVA and Turkey’s post hoc test. *: *p* < 0.05, **: *p* < 0.01, ***: *p* < 0.001, ****: *p* < 0.0001. **D** Heatmap of differentially expressed genes in *Gls*-positive, *Hk2*-positive, double-negative, or double-positive cells compared to all other cells. Expression is an average scaled expression of genes with an adjusted *P* value of < 0.01. **E** GO term enrichment in *Gls*-positive, *Hk2*-positive, double-negative, or double-positive fibroblasts. **F** Expression of selected marker genes in *Gls*-positive, *Hk2*-positive, double-negative, or double-positive fibroblasts from synovial STIA fibroblasts. **G** Expression of selected marker genes in GLS-positive, HK2-positive, double-negative, or double-positive fibroblasts from [25]

We then subset murine fibroblasts and segregated them into double-negative (0 reads for *Hk2* and 0 reads for *Gls*), double-positive (> 0 reads for *Hk2* and > 0 reads for *Gls*), Gls-positive (> 0 G reads), and *Hk2*-positive (> 0 *Hk2* reads) (Fig. 2A) and verified this segregation by examining gene expression of *Hk2* and *Gls* (Fig. 2B). Of interest, the double-positive cells bearing both gene markers increased in number during the peak while the double-negative cells went down (Fig. 2C). Transcriptionally, double-positive fibroblasts showed some overlap in the number of genes expressed by *Hk2*-positive cells and less of genes expressed by *Gls*-positive cells (Fig. 2D) with several genes including *Ptgs2*, *Hif1a*, *Timp1*, *Cxcl5*, and *Plod2* only associated with double-positive fibroblasts. Genes like *Dkk3*, *Bgn*, *Col6A2*, and *Itm2A* were exclusive to *Gls*-positive fibroblasts while *Maff*, *Cdkn1a*, and *Socs3* were unique only to *Hk2* fibroblasts*.* Inflammatory-related genes like *IL-6*, *Cxcl1*, *Ccl7*, and *Myc* were common between *Hk2*-positive and double-positive fibroblasts*.* Moreover, genes associated with the extracellular matrix ECM-like *Col1A2* and *Col1A1* were overlapping between *Gls*-positive and double-positive fibroblasts (Supplementary file 1).GO term analysis showed that *Hk2*-positive cells were more inflammatory associated, double-negative were more associated with negative regulation of cell migration, and double-positive and *Gls*-positive were more associated with ECM modulation (Fig. 2F). In particular, murine double-positive fibroblasts were enriched in *Has1*, *Col12a1*, *and Col3a1*, and double-negative cells were more associated with *Itm2b*, *Angptl7*, and *Igfbp6*. We also segregated the fibroblasts from Zhang et al. (2019) using the same methodology (Supplementary Fig. S1A–C) to confirm our findings (Fig. 2G).

### Modulation of glucose and glutamine metabolism

**Fig. 3:**
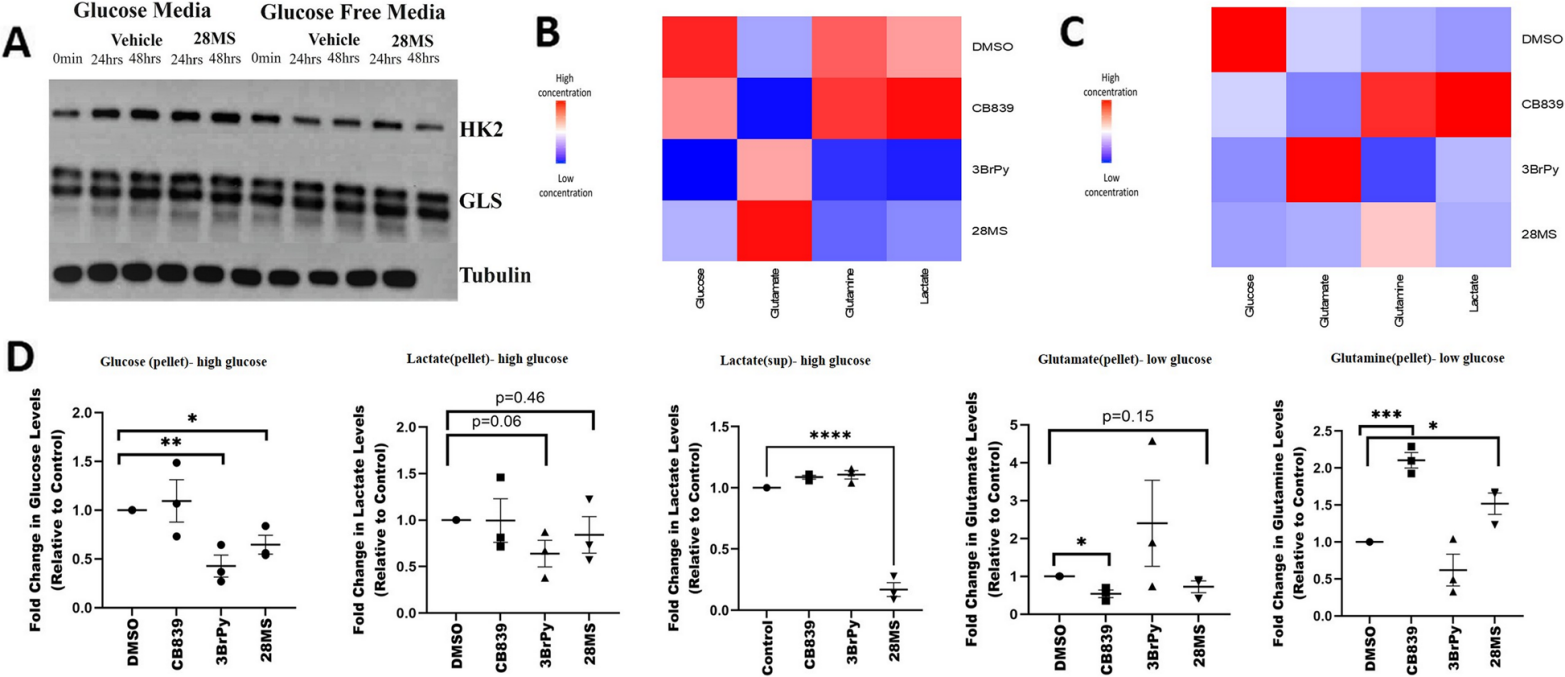
Therapeutic inhibition of glycolysis and glutaminolysis. **A** Representative western blot showing that the expression of HK2 and GLS1 in glucose and glucose-free media remains unaffected by the action of c28MS in RAFLS (*n* = 3). **B** Color scale encoded heat map illustrating variations in the concentration of key metabolites in RAFLS (*n* = 3) between control and treatment groups in glucose medium (glucose medium; 25 mM of glucose and 6 mM of glutamine). **C** Color scale encoded heat map illustrating variations in the concentration of key metabolites in RAFLS (*n* = 3) between control and treatment groups in low-glucose medium (2 mM of glucose and 6 mM of glutamine). **D** One-dimensional H^1^ nuclear magnetic resonance spectrum fold change in glucose and lactate levels in glucose medium (25 mM of glucose and 6 mM of glutamine) and glutamate and glutamine in low-glucose medium (2 mM of glucose and 6 mM of glutamine) of treated and untreated RAFLS (*n* = 3). Values are expressed as mean ± standard error mean. *p* < 0.05 was considered statistically significant

To study the effect of inhibiting both enzymes in RAFLS, we first determined whether our compound inhibits glucose and glutamine metabolism. Western blot revealed that compound 28MS (c28MS) did not affect the expression of target enzymes HK2 or GLS1 in both media with and without glucose, suggesting only potential blockage of target enzymes without affecting their respective expressions (Fig. 3A). Metabolic profiling revealed that both 3-bromopyruvate (3BrPy), a non-specific HK inhibitor, and c28MS were highly effective in reducing the amount of available glucose and consequently lactate for downstream processing to the cells in glucose media. CB839, a glutaminase inhibitor, showed little to no effect in reducing the available amount of glucose to the cells. We also assessed the fold change levels of lactate in the pellet and supernatant and found that the c28MS significantly diminished the lactate levels in the supernatant. In the presence of glucose, CB839, a glutaminase inhibitor, marginally raised the glutamine levels and reduced the glutamate levels. This effect was however not observed by c28MS and 3BrPy in the presence of glucose (Fig. 3B, D). Under low concentrations of glucose, CB839 was highly effective in increasing the glutamine and reducing the glutamate levels in the cells followed by c28MS. In contrast, 3BrPy acted to reduce the glutamine levels and increase the glutamate levels in the cells (Fig. 3C–D). Furthermore, in an abundance of glucose, a general escalation of choline, glycine, and tryptophan because of treatment of 3BrPy and c28MS was observed. Moreover, acetate, betaine, butyrate, cysteine histidine, leucine, isoleucine, methionine, phenylalanine, threonine, tyrosine, and valine were downregulated. However, the effect of c28MS on citrate, alanine, arginine, asparagine, formate, and lysine in the presence of glucose was not synonymous with 3BrPy (Supplementary Fig. S2A). Under low-glucose conditions, treatment with CB839 and the c28MS increased acetate, alanine, betaine, formate, histidine, isocitrate isoleucine, o-phosphocholine, tryptophan, and valine whereas phenylalanine, proline, and tyrosine were reduced. Regulation of other metabolites was affected differently by the action of CB839 and the compound in glucose-free media (Supplementary Fig S2B).

### Inhibiting the glucose and glutamine metabolism by c28MS reduces RAFLS invasion, migration, and proliferation

**Fig. 4:**
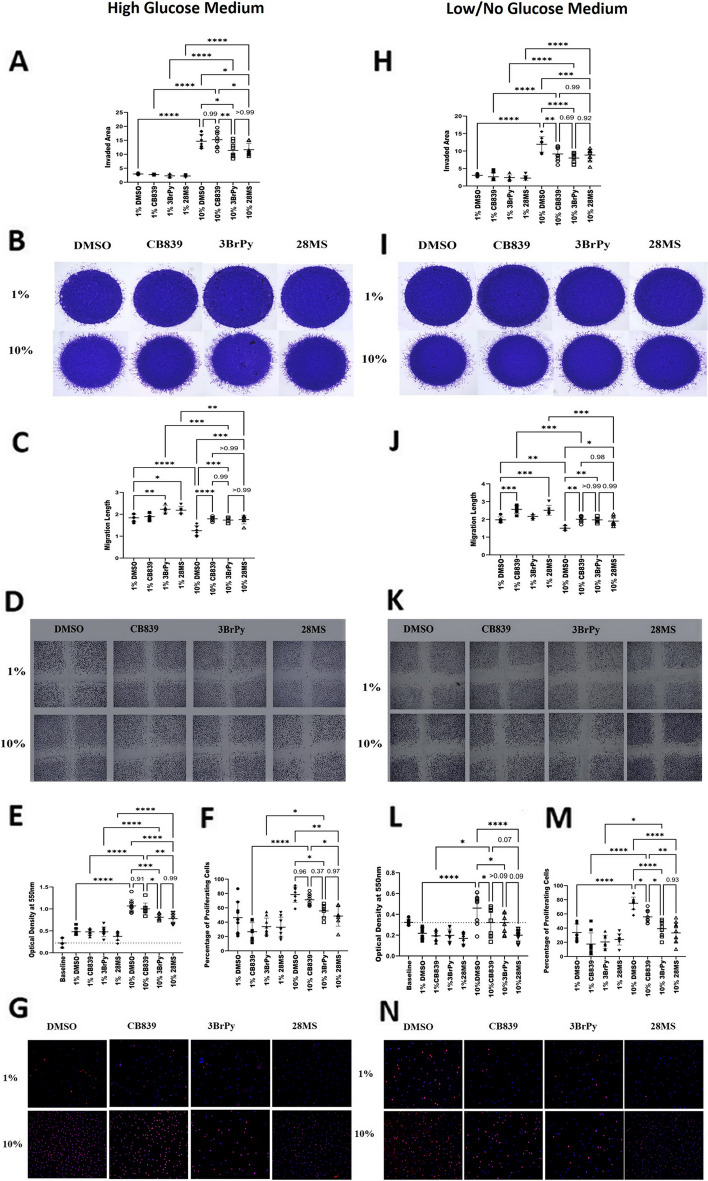
Functional assays to establish inhibitory effects of c28MS and standard treatments in RAFLS in glucose and glucose-free/low-glucose medium (CB839: 300 nm, 3BrPy: 25uM, c28MS: 2uM).** A**–**D**,** H**–**K** RAFLS (*n* = 3) were plated for matrigel invasion and migration scratch assay in glucose and glucose-free medium. Quantification of the results (**A**,** C**,** H**,** J**) and representative images of RAFLS invasion and migration (**B**, **D**, **I**, **K**) are shown. **E**, **L** RAFLS (*n* = 3) were plated and starved overnight with 0.1% fetal bovine serum (FBS) and inhibitors added the following day in high-glucose (25 mM) and low-glucose (2 mM) medium. MTT was added on day 5 after the addition of conditions. RAFLS (*n* = 3) were starved overnight 0.1% FBS after plating. Compound and standard treatments were added in 1% and 10% FBS media under high-glucose (25 mM) and low-glucose (2 mM) and left to proliferate for 4 days in the presence of EdU and consequently counterstained with Hoechst 33,342 (blue) dye to assess the total proportion of cells.** F**, **M** Results were quantified as the percentage of EdU-positive cells among the total number of Hoechst 33,342-positive cells and representative images shown in** G** and** N**. Horizontal lines and error bars show the mean ± SD. * = *p* < 0.05; **** = *p* < 0.0001

Obstructing the elevated glucose and glutamine metabolism itself has been known to diminish the aggressive baneful behavior of RAFLSs by reducing their invading, migrating, and proliferating capability. Our results showed that the cells remained viable at a working concentration of less than 5uM for the compound under study (Supplementary Fig. S3A). Matrigel invasion was used to evaluate the effect of the compound on the invading capacity of the RAFLS in a glucose medium. Our results show that both the c28MS and 3BrPy effectively acted to reduce the escalated invasion of RAFLS in comparison to the control in glucose media. CB839 did little to nullify the invading proclivity of the RAFLS in glucose media (Fig. 4A, B). In the presence of glucose, both 3BrPy and c28MS were effective in reducing the migration of the aggressive RAFLS (Fig. 4C, D). Proliferation is one of the hallmarks of aggressive cells like RAFLS. Inhibition of the proliferating capacity was evaluated by MTT and further attested by EdU Assay. MTT revealed that only the treatment with 3BrPy and c28MS impaired the escalated proliferation of RAFLS under glucose conditions, with c28MS leading to 3BrPy. However, CB839 was not effective in lessening the proliferation under these conditions. A similar activity pattern suggesting a high anti-proliferative effect of 3BrPy and c28MS and limited anti-proliferative effect of CB839 under glucose concentrations was further verified by EdU assay (Fig. 4E, F). Glucose deprivation potentially enhances glutamine metabolism. Blocking glutamine metabolism is known to deescalate the aggressiveness of RAFLS. To further strengthen the efficacy of c28MS as a glutaminase inhibitor, its effect on invasion, migration, and proliferation in glucose-free/low-glucose conditions was assessed. Like our results in medium with high glucose, the cells remained viable at a working concentration of less than 5 μM for the compound under study even under low-glucose conditions (Supplementary Fig. S3B). Under glucose-deprived conditions, both standard treatments CB839, 3BrPy, and c28MS markedly decreased the matrigel invasion and migrative capabilities of the RAFLS (Fig. 4G–J). Of interest, c28MS was more effective in decreasing RAFLS proliferation than CB839 under glucose-deprived conditions (Fig. 4K, L).

### Blocking glucose and glutamine consumption modulates the TNF-induced IL-6, MMP-3, and CCL2 production in RAFLS

**Fig. 5:**
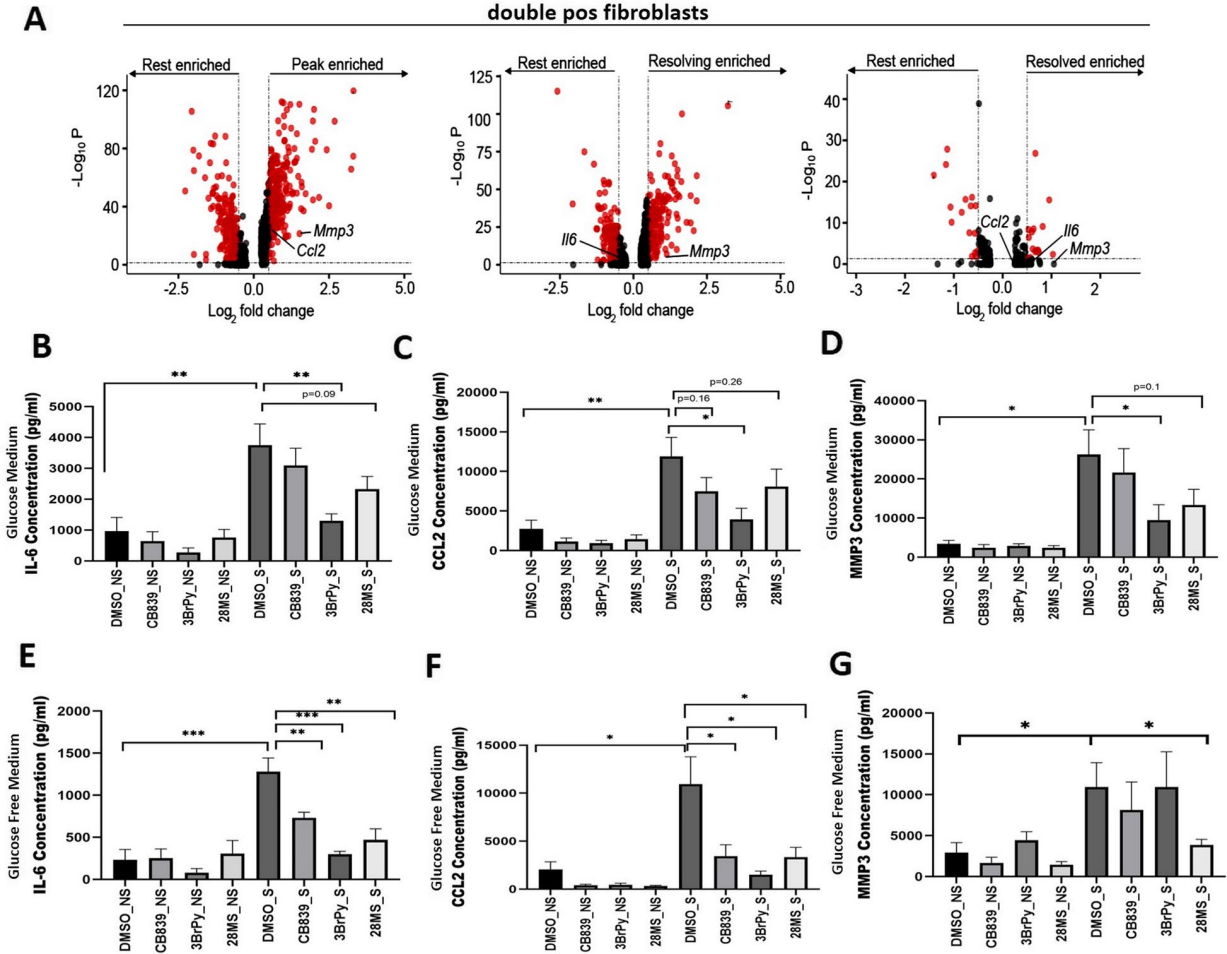
Effect of treatment on the levels of disease markers identified by scRNA-seq.** A** Volcano plots exhibiting the presence of IL-6, CCL2, and MMP3 between the indicated stages of disease in *Hk2*- and *Gls-* (*double-positive*) positive cells. Each dot represents a gene. Genes in red: adjusted *p* value < 0.05 and foldchange > 0.5 and <  − 0.5. *p* value calculated using FindMarkers() (Seurat).RAFLS (*n* = 3) were incubated with vehicle and inhibitors (CB839 (300 nM), 3BrPy (25 μM), and c28MS (2 μM)) for 1 h followed by stimulation with TNF (5 ng/mL) for 24 h. The production of IL-6, CCL2, and MMP-3 in the culture supernatants of RAFLS (*n* = 3) was measured using commercially available ELISA kits in the presence (**B**–**D**) or absence (**E**–**G**) of glucose. Values are mean ± standard error mean. *p* < 0.05 was considered statistically different

The differentially expressed genes in murine STIA double-positive fibroblasts (Supplementary file 2) were examined by comparing peak inflammation (d7-9)/resolving (d15)/resolved (d22) time points to resting (d0). We found that at peak, the expression of *Ccl2* and *Mmp3* were enriched in the double-positive fibroblasts, suggesting that these may be the main effector molecules in these cells that drive inflammation. The enrichment of *Mmp3* continued in resolving and was lost in resolved (Fig. 5A, Supplementary Fig. S4).

Subsequently, taking the detected presence of MMP3, IL-6, and CCL2 in different phases of the diseases, we next aimed to evaluate the effect of our treatments on the levels of these molecules under glucose and glutamine-enriched conditions. In the presence of glucose, 3BrPy was the most effective treatment in significantly reducing the detrimental levels of IL-6, CCL2, and MMP3. c28MS was also able to reduce the strikingly high levels of all three pathogenic markers in RAFLS after TNF stimulation. However, like most of our other results, ensuing to the presence of glucose, CB839 had limited efficacy in reducing the escalated levels of IL-6, CCL2, and MMP3 (Fig. 5B–D). The reduction of both IL-6 and CCL2 was more striking in glucose-deprived conditions as all three treatments significantly diminished the burgeoning levels of both IL-6 and CCL2 (Fig. 5E, F). Moreover, in the absence of glucose, only the c28MS compound worked effectively to reduce the MMP3 levels in comparison to all other treatments (Fig. 5G).

### c28MS significantly decreased arthritis severity in mice

**Fig. 6:**
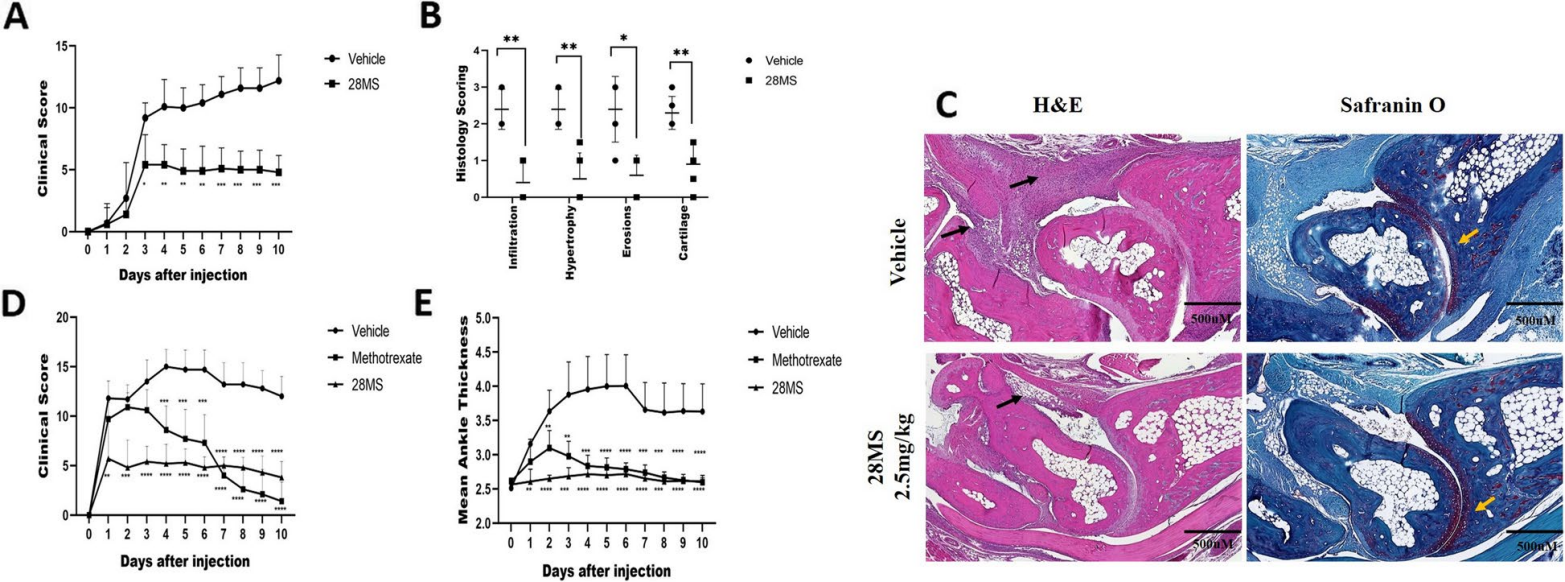
Treatment with c28MS decreases clinical scores and histologic scores in the K/BxN mouse model of arthritis. **A** Clinical scores were determined in vehicle-treated animals (*n* = 5) and c28MS-treated animals (*n* = 5) at a dose of 2.5 mg/kg. One hundred fifty microliters of K/BxN mouse serum was injected at day 0 and treatment was administered intraperitoneally daily from day 0 and continued until day 10. **B** Histologic scores were determined on day 10 after serum transfer in vehicle-treated or c28MS-treated mice. **C** Sections of the ankle joints of vehicle or c28MS-treated mice were stained with hematoxylin and eosin (H&E) or Safranin O on day 10 after arthritis induction. Black arrows indicate joint inflammation and hypertrophy, and yellow arrows highlight cartilage damage, which was reduced in c28MS-treated mouse ankles. **D**, **E** Induction of arthritis and treatment (c28MS and MTX) prognosis in treated and untreated K/BxN mice model (*n* = 5 per group) **D** clinical score and **E** paw swelling. Horizontal lines and error bars show the mean ± SD. * = *p* < 0.05; **** = *p* < 0.0001

The K/BxN serum transfer-induced arthritis (STIA) model is a robust arthritis model that allows the study of the effector phase of RA. As depicted by the decrease in clinical and histological scores in the K/BxN model (Fig. 6A, B) c28MS prevented the development of arthritis. Robust joint inflammation was observed in the control group whereas it was absent in the group treated with the compound. Importantly, treatment with c28MS averted the development of arthritis, and positively repressed joint swelling in the established mice model. The histological score showed that on day 10 of arthritis, the joints exhibited distinctly diminished cellular infiltration and cartilage and bone damage in animals treated with c28MS in contrast to animals treated with vehicle only (Fig. 6C). We also compared the effect of c28MS with methotrexate. c28MS reduced the clinical score right from the beginning of the treatment, whereas methotrexate began to exhibit a significant effect by reducing the clinical score on day 4 (Fig. 6D). Both treatments, however, were quite comparable in terms of decreased paw swelling (Fig. 6E).

## Discussion

Targeting metabolism in cancer cells by dual inhibitors has drawn remarkable attention [26–28]. However, the scope of a similar approach has not been fully explored in RA. Therefore, the undertaken study evaluates the dual inhibition of glycolysis and glutaminolysis using a small drug like the c28MS compound. Based on the data of our study we propose that simultaneous inhibition of these metabolic pathways could complement existing RA treatments by suppressing the pathological behavior of the FLS. Although the molecule might target various cell types, the current study focuses solely on the effect of co-inhibiting HK2 and GLS on FLS.

Dual inhibition is beneficial in complex disorders like RA because the therapeutic regulation of a single pathway may not be as efficient to achieve the desired treatment results. Synergistic inhibition by dual or multi-target agents seems plausible as they can avert the shortcomings of current combination therapies such as side effects, drug interactions, or resistant pathways [29]. Due to the diversity of cellular pathways, exploring the effect of a dual inhibitor in a non-metabolic scenario has been the focus of some studies. The inhibition of targets like SYC and JAK3 or IL-17 and TNF by action of dual inhibitors in RA has been described [30–32]. The anti-inflammatory dual 5LOX-COX inhibitor has also been the focus of many studies [33–35]. Other research has also reported a similar non-metabolic target, dual inhibition approach for diseases like cancer and Alzheimer’s [36–40].

PI3K/Akt/mTOR, JAK-STAT, AMPK, and Wnt/β-catenin pathways have been strongly implicated with cancer metabolism. The PI3K/Akt/mTOR pathway dual inhibitors have exhibited a promising therapeutic effect [41–44] in cancer. Yang et al. reported a dual inhibitor of AMPK and WNT/β-catenin which displayed promising anti-cancer activity against colorectal cancer [45]. The repression of HDAC- JAK and AMPK-MELK by action of dual inhibitors has also proved effective in vitro and in vivo [46, 47]. Despite the reported efficacy of these inhibitors, further research is needed for validation. And like all therapies, this approach has its downfalls as well, such as a lack of predictive biomarkers for the assessment of the treatment’s effectiveness, toxicity, or variance in efficacy according to the type of cancer [41].

In our work, we suggest fibroblasts as specific target cells for the safe and reliable synergistic suppression of glycolysis and glutaminolysis. Targeting fibroblasts would prevent the needless tampering of the immune system and preclude the possible repercussions of global glycolytic and glutaminolytic inhibition. Of interest, HK2 was mostly expressed in macrophages and fibroblasts and showed to be dispensable in T cells, yet GLS1 is crucial in T cell immunity [48–51]. Our study findings elucidate that HK2 and GLS are two distinct pathways that drive various genes involved in vital cellular processes, inflammatory cascades, response to stimuli, ECM components, and remodeling. The results of our study indicate a more prominent expression of HK2 and GLS in sub-lining fibroblast cluster/clusters. The inducible nature of HK2 [23, 52] is further strengthened by our results as the number of HK2-positive cells increases at the peak of the disease in comparison to the baseline/resting phase of the disease. Furthermore, the number of double-positive cells also increases as a result of the inflammation further suggesting HK2 and GLS as players of the pathological metabolism in RA. Thus, obstructing both targets with a dual inhibitor would offer better control of RA.

Additionally, the detection of genes like Ptgs*2*, *Cxcl5*, *Timp1*, and *Hif1a* solely on double-positive fibroblasts also strengthens our concept of synergistic inhibition in RA [8, 53–58]. Other genes such as *Has*, *Postn*, *Plod2*, and *Phlda1* were detected in *Hk2*, *Gls*, and double-positive fibroblasts (Supplementary Table 1). These genes have been linked to the aggressive behavior of cells in RA and other disorders [8, 59–63]. Consistently, c28MS reduced the invasion, migration, and proliferation, regardless of the concentration of glucose in the RAFLS. In addition, the compound also modified the cytokine profile, which was relatively more affected by glutamine metabolism inhibition than glucose metabolism inhibition. The existing literature is heterogenous when it comes to glutamine metabolism affecting the cytokine profile [8, 64] whereas glucose starvation has been known to reduce the amounts of the inflammatory cytokines like IL-6, in addition to degradative enzymes like MMP1 and MMP3 [65].

According to our findings, concurrent expression of HK2 and GLS was observed in many fibroblasts, with each gene governing distinct pathways that lead to the development and subsequent progression in RA. Decreasing glucose and/or glutamine has been shown to be therapeutically important [11, 66]. Our findings indicate that inhibiting both glycolysis and glutaminolysis is more beneficial than solely inhibiting glutaminolysis with CB839. However, we were unable to establish that our compound is superior to 3BrPy, possibly due to 3BrPy’s ability to act on multiple enzymes beyond HK2. Additional targets of 3BrPy include lactate dehydrogenase (LDH), pyruvate dehydrogenase, isocitrate dehydrogenase (IDH), and alpha-ketoglutarate dehydrogenase [17, 18]. However, c28MS and 3BrPy displayed a fair degree of similarity in their ability to inhibit HK2 thereby suppressing glycolysis and c28MS decreased intracellular glucose and lactate levels in the presence of glucose-like 3BrPy. Interestingly, in the presence of glucose, unlike CB839, the capacity of the compound to obstruct extensive glutamine consumption was hindered. However, the capability of the compound to block glutamine metabolism was only accentuated under low-glucose conditions. Although our NMR results showed that CB839 did not reduce the lactate levels in RAFLS, Zacharias et al. reported that obstruction of glutaminase activity by the action of CB839 has been shown to reduce the amount of lactate in AML (acute myeloid leukemia) [67]. Additionally, the CB839 treatment has been shown to reduce glucose and lactate uptake in head and neck cancer [68]. This may indicate that non-tumor cell metabolism is different than cancer cells.

The KxB/N model of passive arthritis is highly reliant on FLS and other cells like neutrophils, macrophages, and mast cells [69]. Histological and clinical scores and paw swelling indicated that the compound successfully suppressed inflammatory arthritis both when injected before the arthritis induction and at the arthritis peak. This implies not only a protective but also a therapeutic effect of the compound, which was similar to the effect of methotrexate, thus providing an alternative treatment for RA [70].

In summary, we report that targeting glycolysis and glutaminolysis can be therapeutically more promising as this may modify potential cross-talk amid FLS and other neighboring adjacent cells inhabiting the synovium in contrast to when either of the pathways is inhibited alone. This could complement the existing therapies for RA thereby improving disease outcomes. The acquired data can further find its extension on other rheumatic disorders.

## Conclusion

Our study results propose that glutaminolytic and glycolytic pathways are upregulated in RAFLS and that dual inhibition of excessive consumption of glucose and glutamine could be an effective approach for the treatment of RA by suppressing the aggressive properties of RAFLS. The suppression of glycolysis and glutaminolysis by the action of a dual inhibitor tends to remain a lesser-explored therapeutic approach for RA. Hence, this data provides a potential priming ground for therapeutic suppression of both glycolysis and glutaminolysis for RA and other rheumatic diseases.

### Supplementary Information

**Additional file 1:** **Figure S1.** A scRNA-seq UMAP of all cells sequenced as described in Zhang et al., 2019 [25]. B,C Segregation of synovial fibroblasts from Zhang et al., 2019 [25] in to GLS positive, HK2 positive, double negative and double positive cells.

**Additional file 2:** **Figure S2.** Color scale encoded heat maps illustrating variations in the concentration of various metabolites between control and treatment groups in RAFLS A glucose medium (25mM of glucose and 6mM of glutamine) and B low glucose medium (2mM of glucose and 6mM of glutamine) respectively.

**Additional file 3:** **Figure S3.** Effect of c28MS on viability of RAFLS (*n*=3) A glucose medium (25mM of glucose and 6mM of glutamine) and B low glucose medium (2mM of glucose and 6mM of glutamine).

**Additional file 4:** **Figure S4.** Expression of *Ccl2*, *Mmp3* and *Il6* in double positive and negative fibroblasts at different stages of the disease.

**Additional file 5.** Details of genes found in double negative, Hk2 positive, Gls positive and double positive fibroblasts in murine STIA.

**Additional file 6.** List of up-and down regulated genes in double positive fibroblasts for the different disease stages.

## Data Availability

The data that support the findings in the study are included in the article/Supplementary material. Further inquiries can be directed to the corresponding author.

## Abbreviations

RA: Rheumatoid Arthritis
RAFLS: Rheumatoid Arthritis Fibroblast-like Synoviocytes
FLS: Fibroblast-like Synoviocytes
DMEM: Dulbecco’s Modified Eagle’s Medium
PBS: Phosphate Buffer Saline
HK2: Hexokinase 2
GLS: Glutaminase
LDH: Lactate Dehydrogenase
PDH: Pyruvate Dehydrogenase
IDH: Isocitrate Dehydrogenase
ELISA: Enzyme-linked Immunosorbent Assay
NMR: Nuclear Magnetic Resonance
EdU Proliferation: (5-Ethynyl-2’-deoxyuridine) Proliferation Assay
MTT: 3-(4,5-Dimethylthiazol-2-yl)-2,5 diphenyltetrazolium bromide
GEO: Gene Expression Omnibus
AUC: Area under the curve
STIA: Serum Transfer-Induced Arthritis
