# Supplementary figures and images for: Dual inhibition of glycolysis and glutaminolysis for synergistic therapy of rheumatoid arthritis
Source: Arthritis Res Ther. 2023 Sep 20;25:176. doi: 10.1186/s13075-023-03161-0 (PMC10510293; doi:10.1186/s13075-023-03161-0)

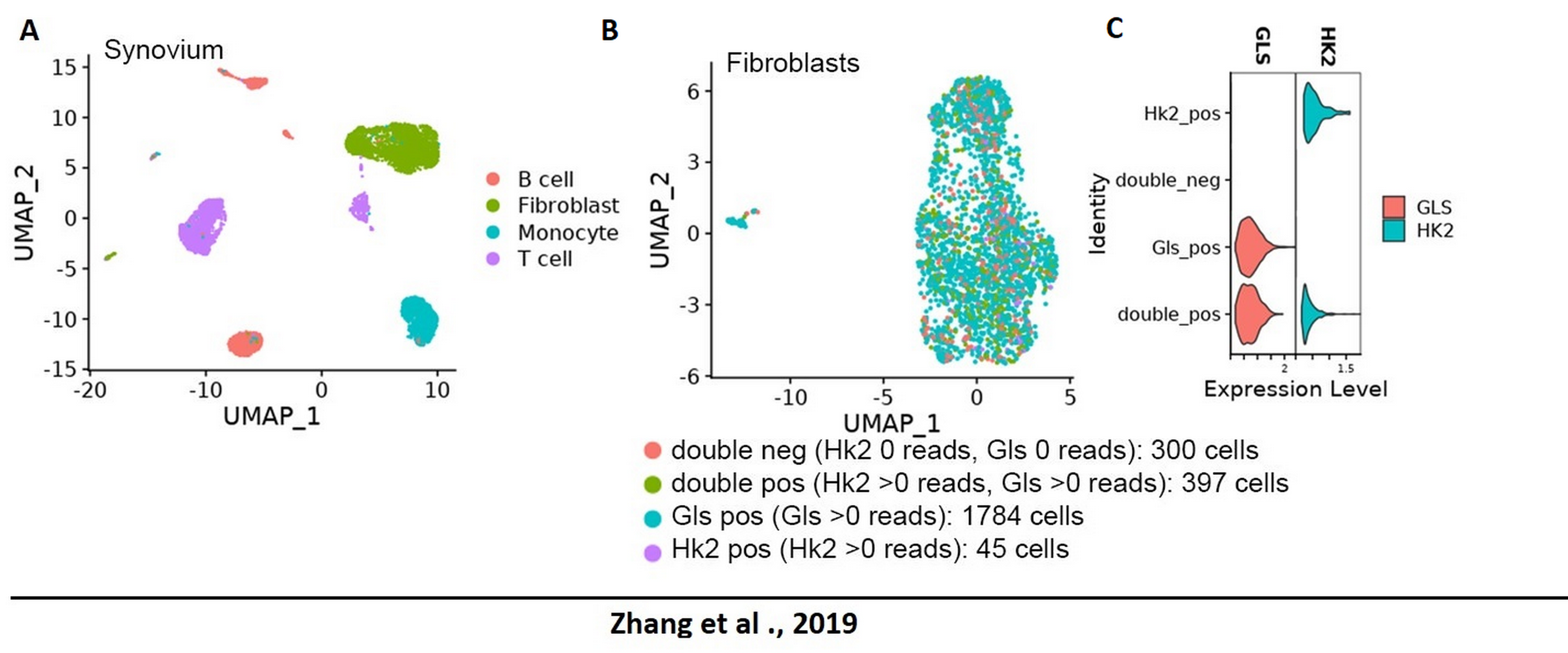

Supplement: Supplementary file 1 — Additional file 1: Figure S1. A scRNA-seq UMAP of all cells sequenced as described in Zhang et al., 2019 [25]. B,C Segregation of synovial fibroblasts from Zhang et al., 2019 [25] in to GLS positive, HK2 positive, double negative and double positive cells. [file 13075_2023_3161_MOESM1_ESM.tif]

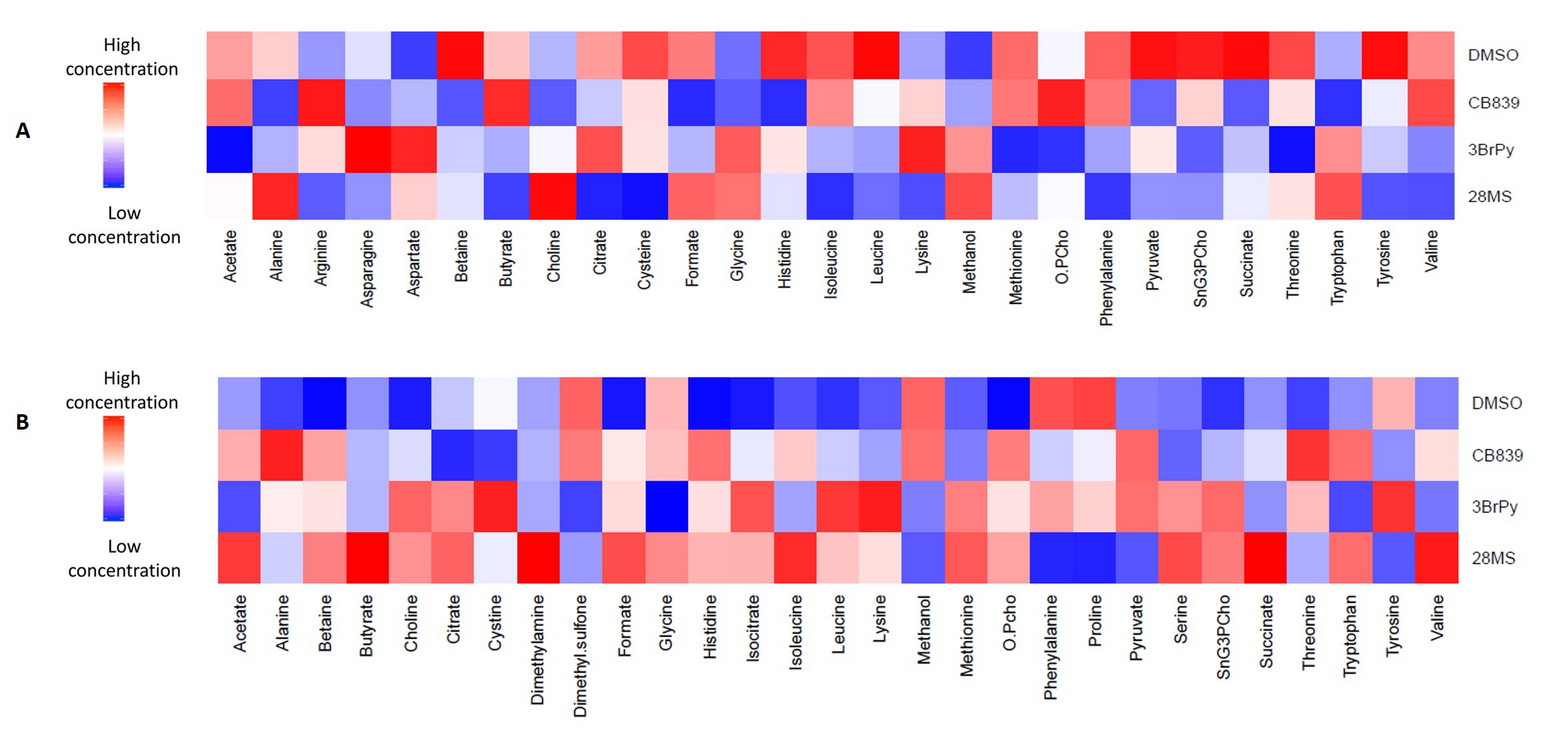

Supplement: Supplementary file 2 — Additional file 2: Figure S2. Color scale encoded heat maps illustrating variations in the concentration of various metabolites between control and treatment groups in RAFLS A glucose medium (25mM of glucose and 6mM of glutamine) and B low glucose medium (2mM of glucose and 6mM of glutamine) respectively. [file 13075_2023_3161_MOESM2_ESM.tif]

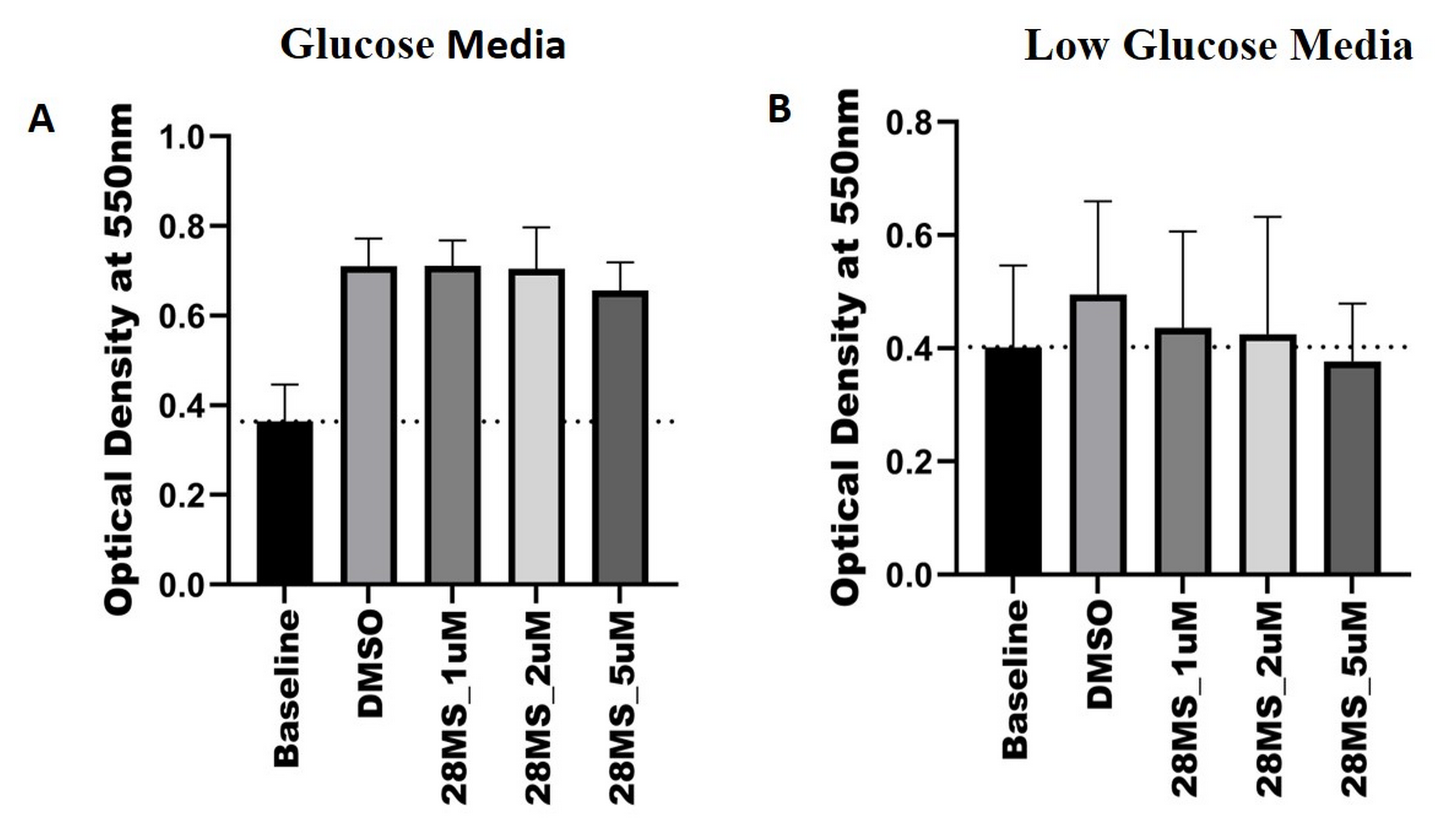

Supplement: Supplementary file 3 — Additional file 3: Figure S3. Effect of c28MS on viability of RAFLS (n=3) A glucose medium (25mM of glucose and 6mM of glutamine) and B low glucose medium (2mM of glucose and 6mM of glutamine). [file 13075_2023_3161_MOESM3_ESM.tif]

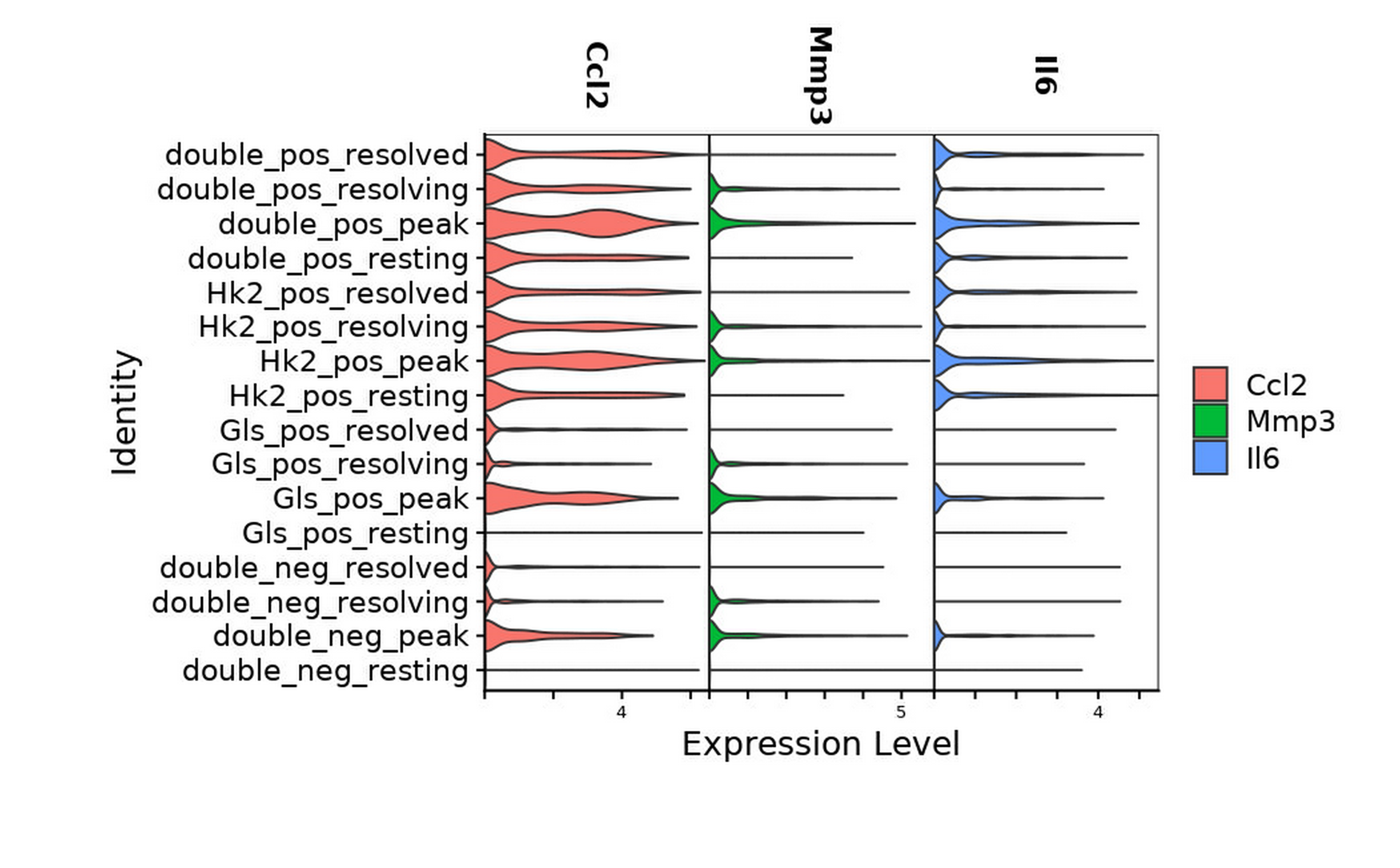

Supplement: Supplementary file 4 — Additional file 4: Figure S4. Expression of Ccl2, Mmp3 and Il6 in double positive and negative fibroblasts at different stages of the disease. [file 13075_2023_3161_MOESM4_ESM.tif]
